# Lipodystrophies—Disorders of the Fatty Tissue

**DOI:** 10.3390/ijms21228778

**Published:** 2020-11-20

**Authors:** Birgit Knebel, Dirk Müller-Wieland, Jorg Kotzka

**Affiliations:** 1 German Diabetes-Center, Leibniz Center for Diabetes Research at Heinrich-Heine-University Düsseldorf, 40225 Düsseldorf, Germany; Birgit.Knebel@ddz.de; 2 Institute for Clinical Biochemistry and Pathobiochemistry, 40225 Düsseldorf, Germany; 3 German Center for Diabetes Research (DZD), 85764 München-Neuherberg, Germany; 4 Clinical Research Center, Department of Internal Medicine I, University Hospital Aachen, 52074 Aachen, Germany; dirmueller@ukaachen.de

**Keywords:** lipodystrophy, generalized, acquired, genetics

## Abstract

Lipodystrophies are a heterogeneous group of physiological changes characterized by a selective loss of fatty tissue. Here, no fat cells are present, either through lack of differentiation, loss of function or premature apoptosis. As a consequence, lipids can only be stored ectopically in non-adipocytes with the major health consequences as fatty liver and insulin resistance. This is a crucial difference to being slim where the fat cells are present and store lipids if needed. A simple clinical classification of lipodystrophies is based on congenital vs. acquired and generalized vs. partial disturbance of fat distribution. Complications in patients with lipodystrophy depend on the clinical manifestations. For example, in diabetes mellitus microangiopathic complications such as nephropathy, retinopathy and neuropathy may develop. In addition, due to ectopic lipid accumulation in the liver, fatty liver hepatitis may also develop, possibly with cirrhosis. The consequences of extreme hypertriglyceridemia are typically acute pancreatitis or eruptive xanthomas. The combination of severe hyperglycemia with dyslipidemia and signs of insulin resistance can lead to premature atherosclerosis with its associated complications of coronary heart disease, peripheral vascular disease and cerebrovascular changes. Overall, lipodystrophy is rare with an estimated incidence for congenital (<1/1.000.000) and acquired (1–9/100.000) forms. Due to the rarity of the syndrome and the phenotypic range of metabolic complications, only studies with limited patient numbers can be considered. Experimental animal models are therefore useful to understand the molecular mechanisms in lipodystrophy and to identify possible therapeutic approaches.

## 1. Congenital Generalized Lipodystrophy

Congenital generalized lipodystrophy (CGL) is usually inherited autosomal recessively and is already observed in newborns. Patients with CGL have a generalized deficiency of fat from birth with a strong muscular appearance (Table 1) [1,2]. Patients usually have severe insulin resistance due to lipodystrophy with pronounced hyperinsulinemia associated with hypertriglyceridemia and reduced HDL cholesterol levels. Diabetes usually manifests itself at puberty and is associated with an enlarged liver and spleen. Acanthosis nigricans occurs later in childhood and young adulthood, usually at typical sites including the neck and armpits [3]. Serum levels of leptin and adiponectin are very low, corresponding to the absence of body fat. Hirsutism, oligo-amenorrhea and polycystic ovaries have been described in girls and women of post pubertal age, respectively.

At present, four subtypes of congenital generalized lipodystrophy (CGL) are distinguished, which are attributable to the genes 1-acylglycerol-3-phosphate O-acyltransferase (AGPAT) 2, Berardinelli-Seip congenital lipodystrophy (BSCL) 2, caveolin (CAV) 1 and caveolae associated protein (CAVIN) 1. Among the large number of cases described, the subtypes CGL1 (AGPAT2) and CGL2 (BSCL2) are the most common variants; of CGL3 (CAV1) and CGL4 (CAVIN1) subtypes, however, only a few cases have been described [4,5,6,7,8].

## 2. Genes in CGL

AGPAT2 is strongly expressed in fatty tissue and alkylates lysophosphatidic acid to phosphatidic acid, a key step in the synthesis of glycerol phospholipids and triglycerides. BSCL2 encodes seipin and is highly expressed in adipose tissue, testes and brain. Seipin appears to be necessary for the fusion of lipid droplets. Mice lacking the seipin gene develop dyslipidemia, insulin resistance and hepatic steatosis in addition to lipodystrophy of the white adipose tissue [9,10]. However, the pathogenetic relationships between mutations in this gene and the clinical phenotype are not yet sufficiently understood. Interestingly, patients with CGL2 are more likely to have impaired mental development compared to patients with CGL1. On the other hand, patients with CGL1, in contrast to patients with CGL2, still have mechanical fat deposits (palms of the hands, soles of the feet, orbit, under the scalp and periarticular).

Caveolin 1 (CAV1), which stands for the subtype CGL3 [7], is important for the function of the caveolae, a special form of the lipid rafts of the plasma membrane. Caveolae are invaginations of the plasma membrane involved in endocytosis, signal transmission, lipid transport including the formation of intracellular fat droplets and fat storage. CAVIN1, which causes CGL4, formerly known as polymerase I and transcript release factor (PTRF), is an essential factor in the biogenesis of caveolae. In regard to pathophysiology, the skeletal and cardiac muscles are also affected, so that in these patients not only lipodystrophy but also muscular dystrophy and long QT syndrome with sudden cardiac death can be observed [4,10].

In addition to these known as CGL in OMIM (Table 1), generalized lipodystrophy can also occur in other Mendelian diseases such as insulin resistance syndrome and complex progeroid syndrome (see below). A de novo variant in the promoter of the c-fos gene [11] is an example of how complex the molecular relationships of CGL are. This gene codes for FOS, a protein of the immediate-early gene family, which plays a central role as a primary response gene, for example in fat cell differentiation.

## 3. Partial Congenital Lipodystrophy

In contrast to CGL, patients with familial partial lipodystrophy (FPLD) do not have a generalized lack of fat, but are characterized by a lack of fat in the area of the limbs, buttocks and hips (Table 2) [1,2,12,13,14,15,16,17,18,19,20,21,22]. Those affected have a normal fat distribution at birth and during childhood, but lose subcutaneous fatty tissue on the extremities and various parts of the trunk at the beginning of puberty, resulting in a more “muscular” appearance. Interestingly, women are more affected by metabolic disorders than men. For example, over 50% of women develop diabetes mellitus compared to about 20% of men. The insulin-mediated glucose uptake is greatly reduced, and there are significantly increased levels of free fatty acids in plasma. In addition, acanthosis nigricans, hirsutism, irregular menstrual cycle and polycystic ovaries are found in about 20–35% of patients.

## 4. Genes in FPLD

The type Dunnigan (FPLD2), the most common type of FPLD, is due to molecular changes in the lamin A/C (LMNA) gene. The laminae form hetero- and homodimeric structures of the nuclear lamina and form polymeric intercalated structures between chromatin and the inner membrane of the nuclear envelope. Most missense mutations were found in exon 8, which codes for the globular N-terminal region of the protein. Mutations in this gene have also been reported in several other diseases, such as dilated cardiomyopathy and various forms of muscular dystrophy. The reduced number of fat cells in these diseases could be due to an altered interaction of the lamina with chromatin, which in turn leads to altered cell division and possibly increased apoptosis and cell death. In the Köbberling type variant (FPLD1), which is pathophysiologically limited to the loss of fatty tissue of the extremities, the causative gene is unknown. However, on the basis of clinical evidence, it can be assumed that this type is a variant of the Dunnigan type. Variants of type FPLD3, a rather stereotypical form of lipodystrophy of type PPARγ ligand resistance syndrome (PLRS), are due to heterozygous mutations in the peroxisome proliferator-activated receptor γ (PPARγ) gene. This protein is an important transcription factor in adipocyte differentiation. The variants of type FPLD4 are caused by the protein perilipin 1 (PLIN1) and of type FPLD5 by the protein cell death-inducing DNA fragmentation factor C (CIDEC), both of which play a role in fat droplet formation. Adipocytes have one central lipid droplet with a high surface/content ratio that is crucial in the regulation of lipid release. In contrast, other cell types that store lipids in plenty of small fat droplets. Perilipins like PLIN1 are scaffold proteins that coat lipid storage droplets and regulate lipolysis [23]. This is protective against lipolysis of lipids from the fat droplets by lipase (ATGL) and hormone sensitive lipase (HSL) coded by the LIPE gene [24,25]. Plin1 is thus involved in the storage and stabilization of lipids in a form that is chemically inert for the cell. The loss of function of PLIN1 e.g., in FPLD4 leads to partial lipodystrophy, especially of gluteofemoral and subcutaneous fat of the lower extremities with hypoadiponectinemia, hyperinsulinemia, NAFLD and hypertriglyceridemia [19]. The primary cause appears to be the increased unregulated release of lipid into the cell and the resulting lipotoxicity. This is sufficient to develop a metabolic syndrome. The formation of the adipocyte specific large monocular lipid droplets is regulated by CIDEC [26]. CIDEC mutations as in FPLD5 are similar to FPLD4 in terms of fat distribution, show severe diabetes with ketoacidosis, insulin resistance and hepatic steatosis and multilocular lipid drops in adipocytes [17].

The variants FPLD6 are based on mutations in the gene of hormone sensitive lipase (LIPE) and FPLD7 in the caveolin gene (CAV1). Akt2-coupled lipodystrophy, described in one family, is due to an autosomal dominant mutation in AKT2, a central protein in the insulin-mediated signalling cascade (Table 1 and Table 2).

## 5. Rare Diseases with Familial Partial Lipodystrophy

Familial partial lipodystrophy with mandibuloacral dysplasia (MAD), on the other hand, is a rare autosomal recessive disease that is due to mutations in the lamin A/C gene (LMNA) and mutations in the zinc metalloproteinase gene (ZMPSTE24) [27,28]. ZMPSTE24 processes lamin A from pre-lamin A. In the absence of this protease, a precursor of lamin A that is toxic to the cells accumulates, a defect that has also been observed in progeria syndromes (see below). Patients are conspicuous for changes in the rows of teeth, a bird’s face with protruding eyes, a curved nose, progressive osteolysis of the collarbones and phalanges (Table 1) [1,2].

The rare SHORT syndrome with both autosomal recessive and autosomal dominant inheritance (Table 2) is due to a defect in the subunit of p85 phosphatidylinositol 3-kinase (PIK3R1), a central protein in the cellular signal extension of insulin [29]. The clinical signs of SHORT syndrome are a “short” stature, hyperextensibility of the joints, inguinal hernias, a so-called Rieger anomaly (iris and tooth abnormalities) and delayed dentition. A typical sign is lipodystrophy of the face, upper limbs and trunk. The lower extremities are usually left out. Some of these patients develop insulin resistance and diabetes.

The Wiedemann-Rautenstrauch syndrome, a neonatal progeria syndrome, was first described by Rautenstrauch and colleagues in 1977 and then by Wiedemann in 1979 [30] (Table 2). The inheritance is autosomal recessive and is caused by a defect of the POLR3A gene. The clinical characteristics of this very rare syndrome include a progeroid face, an almost total deficiency of subcutaneous fat, with the subcutaneous fat preserved in the gluteal region. Hutchinson-Gilford progeria syndrome (HGPS) is also characterized by lipodystrophy. This variant is often caused by a single nucleotide polymorphism (C1824T) in the lamin A/C gene, which leads to an accumulation of mutated pre-lamin A, called progerin [31].

## 6. Acquired Generalized Lipodystrophy

Acquired generalized lipodystrophy (AGL) is a rare disease characterized by a loss of fatty tissue during childhood and adolescence. Women are affected about 3 times as often as men. Children can have an increased appetite and increased linear growth. Acromegaloid signs are rarely seen, unlike CGL. Acanthosis nigricans is found in about one third of patients. Most patients have hepatomegaly due to hepatic steatosis, which can also progress to steatohepatitis and cirrhosis. Irregular menstrual cycles and polycystic ovaries can be observed.

The disease was subclassified into AGL type 1 (panniculitis form), AGL type 2 (autoimmune disease) and AGL type 3 (idiopathic form) based on the main clinical manifestations [32]. In the acute form of AGL type 1, sensitive and inflammatory subcutaneous nodules may develop, which, once healed, cause localized atrophy of the subcutaneous fat and then finally generalized lipodystrophy. Pathomorphologically, this type is characterized by an infiltration of the fatty tissue with lymphocytes and histiocytes with a granulomatous reaction and giant multinuclear cells. Patients with AGL type 2, on the other hand, are often affected by other autoimmune diseases such as juvenile dermatomyositis, Sjörgen syndrome, chronically active hepatitis, Hashimoto’s thyroiditis and juvenile rheumatoid arthritis. Therefore, the current pathogenetic notion is that the destruction of adipocytes may be the result of a cell- or antibody-mediated autoimmune process. The pathogenesis of diabetes mellitus is mainly associated with severe insulin resistance. However, in some patients, diabetes mellitus may develop before the onset of lipodystrophy and is then more likely to be type 1 diabetes. The patients need a high dose of insulin.

## 7. Acquired Partial Lipodystrophy

In Barraquer-Simon syndrome (OMIM: #608709), an acquired partial lipodystrophy, the disease usually occurs in children and adolescents, with women affected four times as often as men [33]. Serum complement C3 levels are reduced [34] and polyclonal immunoglobin G or complement 3 nephritic factor (C3NeF) is found in about 90% of patients. This could also contribute to the development of mesangiocapillary glomerulonephritis, which occurs in about 20–35% of patients approximately 10 years after diagnosis. Fat loss usually affects the upper half of the body. Insulin resistance with dyslipidemia and diabetes is not usually found.

Partial lipodystrophy is also found in HIV-infected patients when they are subjected to highly active anti-retroviral therapy (HAART) involving protease inhibitors (PI) or nucleoside reverse transcriptase inhibitors (NRTI) [35,36,37,38]. The clinical appearance is usually characterized by a loss of subcutaneous fat on the face (sunken cheeks) and increased musculature of the extremities with increased fat deposition on the chin (double chin), neck (buffalo neck) and upper trunk. Insulin resistance is usually associated with hypertriglyceridemia. However, patients also develop increased intra-abdominal or visceral fat. HIV patients with lipodystrophy have a normal immune status, a suppressed HIV viral load and no AIDS-defining signs of disease. One hypothesis is that PI and NRTI increase both the differentiation of visceral adipocytes and the apoptosis rate of subcutaneous adipocytes. This can be caused by impaired adipocyte differentiation via SREBP-1, mitochondrial dysfunction in adipocytes, as well as adipocyte apoptosis due to increased pro-inflammatory cytokines such as tumor necrosis factor (TNF)- and IL-6 and the dysregulation of sex hormones. Mechanisms of lipohypertrophy include local increase of cortisol. This can be due either by production or reduction of plasma dehydroepiandrosterone sulfate (DHEAS) to cortisol by dysregulation of 11-hydroxysteroid dehydrogenase (11-HSD), or increased norepinephrine levels [39]. The syndrome occurs in about 15–20% of patients within the first year of HAART and affects almost half of the patients on prolonged therapy with PI. This makes it probably the most common form of lipodystrophy at the moment.

## 8. Therapeutic Approaches for Lipodystrophy

Similar to obesity, therapy in lipodystrophy should aim to reduce adipocyte overload with lipids of the remaining fat depots, or to reduce ectopic lipid load in non-adipose tissues. To achieve this goal, attempts to reduced dietary energy uptake, increase adipocyte number, or bypass the systemic complications due to the fat loss may be promising. The loss of fat is associated with reduced concentrations of the metabolic regulatory adipokines leptin and adiponectin. Consequently, the substitution of leptin was the first therapy to improve metabolism and quality of life tested in studies of both congenital and acquired lipodystrophies [40]. Metreleptin, a recombinant analogue of human leptin, is the only specific therapy available for the management of human lipodystrophies that improve insulin sensitivity [41,42,43,44]. Substitution of the second central adipokine adiponectin is not trivial. It is extremely difficult to synthesize biologically active adiponectin due to the complex posttranslational modifications [45]. Adiponectin analogues have not yet been systematically investigated in lipodystrophies. Alternatives to substitution would be the triggering of endogenous levels of adiponectin by glitazones. These substances act as PPARγ agonists and increase the adiponectin concentration [46]. PPARγ itself is crucial for differentiation of remaining preadipocytes to adipocytes and may thus increase adipocyte numbers. Therefore, in cases of lipodystrophy as a consequence of HAART, glitazones are a successful approach, as well as the combination of leptin and glitazones [47], even though a positive effect on glucose metabolism is not clear [48].

The major approach still remains to address the metabolic consequences of lipodystrophy such as insulin resistance, hyperlipidemia and ectopic lipid accumulation with conventional therapies. This also includes the control of hyperglycaemia with insulin sensitizers. Metformin is the first-line therapy although some patients may require additional agents including high doses of insulin. In individual patients with FPLD insulin secretion disruption [49] or low dipeptidyl peptidase 4 (DPP4) levels were observed in addition to insulin resistance, suggesting the use of glucagon-like peptide 1 receptor agonists (GLP1) [50] to improve glycemic control and reduce the high insulin demand. However, no studies in the other forms of lipodystrophy have yet been conducted [51]. Furthermore, SGLT2 inhibitors have been used in individual families of congenital lipodystrophy [52]. A systematic investigation has not yet been performed to our knowledge. Nevertheless, weight management, caloric restriction and even bariatric surgery can be effective in FPLD patients.

Due to the rarity of the syndrome and the phenotypic range of metabolic complications, only studies with limited patient numbers have been considered. Efforts, such as the collection of cases in databases e.g., TuLip, are invaluable to bring a systematic approach to the clinical manifestations of different lipodystrophies and possible therapies [53].

## 9. Use of Animal Models to Determine Molecular Mechanisms in Lipodystrophy

Although some candidate genes and their function have been identified, the pathogenesis of insulin resistance, hyperlipidemia and abnormal fat distribution in congenital lipodystrophy syndromes is largely unknown. Laboratory animals can be used to gain further knowledge on the molecular processes.

The genetic basis of CGN and FPLD can essentially be divided into two groups. Genes that play a crucial role in the differentiation and maintenance of the function of adipose tissue like, BscL2, PPARγ, AGPAT2, and LMNA, or genes involved in the formation of lipid droplets like PLIN-1, and CIDEC.

Seipin (BscL2) was the first gene identified in patients with the extreme congenital Berardinelli-Seip-Lipodystrophy. BscL2 is an integral homo-oligomeric membrane protein in the endoplasmic reticulum that plays a role in the maturation of cytoplasmic lipid droplets Bscl2^−/−^ mice develop severe white adipose tissue lipodystrophy, dyslipidemia, insulin resistance and hepatic steatosis. In embryonic fibroblasts of these animals, the first steps of adipocyte differentiation are regular, but terminal differentiation into mature adipocytes is disturbed. At the same time, massive activation of lipolysis by cyclic AMP-(cAMP)-dependent protein kinase A (PKA) was observed. Furthermore, expression of the transcription factors of the differentiation cascade and fat tissue specific transcription factors was repressed [10,54]. Solely inhibition of lipolysis can reverse this process. Seipine interacts with lipid homeostasis and limits the formation of fat droplets in non-adipocytes or increases the storage capacity in adipocytes. The influence on the metabolic state is probably regulated by the degree of lipotoxicity.

In the development of adipose tissue, adipocyte precursors from mesodermal precursor cells are differentiated into mature adipocytes by sequential transcription factor activation [55]. A hallmark of adipocyte precursors is the expression of the PPARγ determines the complete differentiation process and is necessary to maintain differentiation. Mutations that alter the function of PPARγ thus directly influence fat cell differentiation. At the same time PPARγ is regulated by lipids. Homozygous PPARγ knockout (KO) mice are not viable [56]. Mice with heterozygous, tissue-specific or conditional PPARγ KO are lipodystrophic with insulin resistance and dyslipidimia [57,58,59]. Consequently, the phenotypes resulting from mutations of a thrifty gene like PPARγ should be rather severe. In CGL cases, usually homozygous mutations or compound heterozygous variants are present [15]. In FPLD3, heterozygous variants of PPARγ are described in functionally relevant regions such as the DNA or the ligand binding domain. These lead to an earlier and more extensive loss of adipose tissue with a corresponding metabolic phenotype and severe insulin resistance and dyslipidemia compared to other FPLDs. Interestingly, hypertriglyceridemia is more dependent on high fat intake [60]. However, also from investigations of animal models, it is not clear how the typical patterning of fat distribution occurs.

AGPAT2 is central in the synthesis of triglycerides from glycerol-3-phosphate. In animal models, Agpat2^−/−^ leads to loss of white and brown adipose tissue and organomegaly. The latter is due to chronic activation of insulin-like growth factor (IGF)-1 and -2 receptors in the liver of these animals. Extreme hyperinsulinemia, diabetes, insulin resistance and hepatomegaly develop in animals already at the age of 2–3 weeks [61,62,63]. Interestingly, other AGPATs do not seem to be able to compensate for the loss of AGPAT2 in function, but GPATs (glycerol phosphate O-acyltransferase), which utilize glycerol-3-phosphate instead of acyl glycerol-3-phosphate, are increased. This may cause the probably central disturbance of the phospholipid synthesis, which results in reduced phosphatidylinositols and increased lysophosphatidylcholines. The loss of function in early adipogenesis is through a network of mechanisms. Here, premature apoptosis of adipocytes is disturbed via Akt signaling. In addition, substrate inhibition of PPARγ occurs by the differential abundance of lipid agonists [64], thus directly affecting adipocyte differentiation. In the Agpat2^−/−^ mouse the de novo lipid synthesis (DNL) is additionally increased. As a consequence, comparable to the lipodystrophy models A/ZIP and aP2-SREBP-1c [65,66], hyperinsulinemia, hyperglycemia and hepatic steatosis occurs here, even if the mechanism here is not directly mediated by SREBP-1. PPARγ overexpression e.g., by leptin can constitute the adipogenic potential in Agpat2^−/−^ knockout, as well as fat restriction in diet [63].

The LMNA gene encodes lamin A and C which provide structural stability of the nuclear envelope and interact with the cytoskeleton. LMNA mutations probably act structurally by polymerization in the nuclear membrane, which leads to the accumulation of cytotoxic immature proteins, the unfolded protein response, and thus to premature apoptosis. The proteolysis of prelamin A to mature LMNA is controlled by the ZMPSTE24 gene and ZMPSTE24 mutations also lead to unfolded protein response. LMNA are ubiquitously expressed and gene mutations are associated with at least 14 different diseases. Animal models that are either LMNA deficient or express single mutant LMNA forms are essentially models for progeria and age-related metabolic complications [67]. FPLD 2 patients with LMNA mutants show the loss of adipose tissue relatively late and other organs may also be affected in their function. This is more likely to correlate with laminin function in premature apoptosis. The fat cells or depots that are still functional can store fat, which leads to disproportionate fat distribution. LMNA interacts directly with DNA-bound SREBP-1 in vivo. This is probably a negative feedback mechanism leading to inhibition of the transcriptional activity of SREBP-1 [68,69]. It is expected that besides SREBP-1, other transcription factors such as e.g., pRb, which are also involved in adipocyte differentiation, will be regulated in a similar way [70]. The expression of FLPD2 mutation reduces the LMNA SREBP-1 interaction and upregulates SREBP-1 target genes in lipid metabolism [71]. This leads to an endogenous activation of lipid metabolism, which subsequently causes hepatic and finally systemic insulin resistance and hepatic lipid accumulation [72,73,74]. Another link to metabolism is provided by the mTOR signaling pathway. The mTOR signaling pathway is the main regulator of metabolism, growth, proliferation and autophagy in all eukaryotic cells [75]. mTOR is a central pathway of insulin signaling and a link from LMNA to glucose metabolism [76]. Furthermore, mTOR regulates lipid synthesis via SREBP and lipin. In LMNA KO mice, mTOR pathway is overactivated [77], which is reversible by rapamycin. The mTOR pathway is an integrator of environmental influences such as insulin signaling via PI3K and AKT, where negative feedback through Akt phosphorylation leads to degradation of LMNA, which may be disturbed in mutated LMNA.

In contrast to other candidate genes for lipodystrophy, perilipin is almost exclusively expressed in adipocytes. Perilipin-1 (Plin1) deficient mice (plin^(−/−)^) have an inconspicuous phenotype. However, the lean mass is higher and the adipocytes are smaller and denser. Adipocytes of Plin^(−/−)^ mice show an constitutive lipolysis due to the loss of the protective function of PLIN1. In addition, these mice do not exhibit a complete lipodystrophic phenotype and adult Plin^(−/−)^ mice had normal plasma glucose in combination with an increased beta-oxidation of lipids, reduced basal hepatic glucose production and develop late onset peripheral insulin resistance [78,79,80].

Similar to PLIN1-1, CIDEC is another lipid drop-associated protein that is specifically expressed in white adipose tissue. The latter corresponds to the phenotype of Fsp27 deficient mice (fsp27^(−/−)^), the murine homologous of CIDEC. FSP27 is regulated by PPARγ. The fsp27^(−/−)^ mice are protected against diet-induced obesity, have low plasma glucose levels and a higher glucose tolerance. This is possibly related to the increased metabolic rate and by browning of adipose tissue fsp27^(−/−)^ mice. However, over time high-fat diet also induces insulin resistance and hepatic lipid accumulation even in FSP27 deficiency. White adipocytes of these animals uniformly show significantly reduced fat content and decreased TAG storage capacity with multilocular lipid droplets, which may lead to lipid accumulation with corresponding metabolic consequences in other tissues [81].

Overall, these animal models of identified lipodystrophy candidate genes show severe phenotypic changes in addition to lipodystrophy. A paradigm shift in modern molecular medicine is the network-centric approach to gene regulatory events. This approach is especially valuable in the understanding of altered transcription factor activity. In this regard, a lipodystrophy phenotype could be observed in mice solely by tissue-specific expression of metabolically central transcription factors. So, a decisive step in the understanding of adipose tissue differentiation and thus the development of lipodystrophy is based on an animal model with an artificial transcription factor [65]. The tissue-specific expression of the dominant negative transcription factor A-ZIP/F under the control of the adipocyte fatty acid-binding protein (aP)2 promoter leads to lipodystrophy in a mouse model [65]. This phenotype, which is not due to the mutation of a single specific gene, provides deeper insights into the regulatory network of adipose tissue. Mechanistically, it prevents the DNA binding of B-ZIP transcription factors from the C/EBP and AP1 family including proto-oncogene c-Fos, which are important factors in fat cell differentiation. Since A-ZIP/F ultimately interferes with the differentiation cascade of adipocytes at different stages, the mice lack white adipose tissue (WAT) but also brown adipose tissue (BAT). This is phenotypically associated with ectopic lipid accumulation and systemic insulin resistance. Furthermore, it was recently shown in this model that the number of embryonic adipocyte progenitor cells alone is decisive for the WAT mass in the adult animal [82]. There is a direct relation of this artificial model to clinical lipodystrophy. We identify a mutation in the c-fos promoter, a gene of the AP-1 transcription factor family, in a patient with congenital generalized lipodystrophy, hepatic steatosis, severe dyslipidemia, and insulin resistance. The mutation leads to an altered DNA/protein interaction at the c-fos promoter and thereby reduced gene expression of c-fos, thus interfering with the adipocyte differentiation in the patient [11].

Another example of ubiquitous gene expression changes in the pathogenesis of lipodystrophy is the knock-down of the MED19 subunit of the mediator transcription complex. MED19 is involved in the development and maintenance of WAT but not of BAT in a cell model. Under systemic conditions in a mouse model, the adipocyte-specific MED19 knock-down in adipose tissue leads to lipodystrophy, hepatic steatosis and insulin resistance which is associated with a change in the PPARγ-mediated gene expression, but also with beiging of BAT [83].

Next to AP-1 complex proteins and PPARγ, transcription factors of the sterol regulatory element-binding protein (SREBP) family regulate adipocyte differentiation and lipid metabolism. SREBP proteins are a hallmark of isoform and tissue specific differential gene expression networks in lipid metabolic processes [84]. The function-giving protein domain of SREBPs is the basic helix loop helix leucine zipper (bHLH-LZ) which mediates homo- or heterodimerization with other HLH proteins [85]. SREBPs are embedded in an inactive form in the membranes of the endoplasmic reticulum and the nuclear envelope are released by a cholesterol-regulated proteolytic mechanism [84], whereas SREBP-1a activates global lipid synthesis in fast-growing tissues, while SREBP-1c plays a role in the nutritional regulation of lipid metabolism, especially in lipogenic tissues such as the liver or fatty tissue.

Further investigations with transgenic mice underline the specificity of SREBP-1c compared to the isoform SREBP-1a [86]. Transgenic animals expressing the active domain of SREBP-1a under the control of the phosphoenolpyruvate carboxykinase (PEPCK) promoter (the PEPCK promoter is active in the liver, kidney, and adipose tissue) show a significant reduction of WAT depots in addition to the massive fatty liver. Nevertheless, these animals do not develop the specific adipose tissue lipodystrophy phenotype [87]. In contrast, in animals expressing the active domain of SREBP-1c under the control of the PEPCK promoter, have a marginally enlarged liver, and the adipose tissue is unaffected [88]. However, animals that overexpress the active domain of SREBP-1a under the control of the aP2-promoter in adipose tissue show increased fatty acid synthesis in the adipocytes, which leads to hypertrophy of WAT and BAT. The accompanying increased fatty acid release leads to a mild fatty liver in these animals. The adipose tissue of mice with target disrupted SREBP-1, if they are viable, is normal [89].

The probably most physiological mouse model of generalized lipodystrophy is caused by the adipose tissue specific expression of the SREBP-1c under the control of the aP2-promoter. AP2-SREBP-1c mice develop congenital generalized lipodystrophy with insulin resistance-related hyperglycemia and massive fatty liver with significantly elevated plasma triglyceride levels [66,72]. In addition, it was shown that the leptin levels in serum clearly correlated with the reduction of adipose tissue and that energy homeostasis is regulated accordingly in the aP2-SREBP-1c model. When leptin is administered to these animals, the metabolic disorders are corrected and also the liver fat content is reduced, but lipodystrophy is not corrected [90]. Together with the observation that a dominant negative form of SREBP-1c inhibited adipocyte differentiation [91], one might speculate that the disturbance in adipocyte differentiation in analogy to the observations in A-ZIP mice is caused by imbalance in protein abundance of members of HLH-proteins.

## 10. Perspectives

Elucidation of clinical subtypes and genetic background of patients with lipodystrophies and detailed mechanistical analyses in experimental animal models is a paradigm example of bed to bench-side medicine. These analyses paved the way to new insights in our understanding of the role of fat tissue, fat partitioning and has implications for the understanding of the pathogenesis of insulin resistance, diabetes and atherosclerosis. This might lead to new treatment options in the future, not only for patients with lipodystrophies, but also more common forms of fat-related cardiometabolic diseases.

## Figures and Tables

**Table 1:** Classification of autosomal recessive forms of lipodystrophy.

Type	Pathophysiology	Clinical Appearance	Phenotype (OMIM)	Gene (OMIM)
Congenital generalized lipodystrophy (CGL)	AGPAT is a key enzyme in triglyceride and phospholipid biosynthesis.	Lack of fatty tissue at birth.	CGL1 (#608594)	AGPAT2 (#603100)
	Seipin/BSCL2 plays a role in fat droplet formation.	Absence of fatty tissue at birth; cardiomyopathy.	CGL2 (#269700)	BSCL2 (#606158)
	Caveolin is an integral part of the caveolae	Lack of fatty tissue at birth; dwarfism.	CGL3 (#612526)	CAV1 (#601047)
	Cavin is involved in the biogenesis of the caveolae	Absence of fatty tissue at birth; cardiomyopathy.	CGL4 (#613327)	CAVIN1 (#603198)
Mandibuloacral dysplasia (MAD)	Lamin A and C are nuclear lamina proteins.	Absence of the subcutaneous fatty tissue of the extremities.	MADA (#248370)	LMNA (#150330)
	ZMPSTE24 processes pre-lamin A into lamin A.	Generalized loss of fat.	MADB (#608612)	ZMPSTE24 (#606480)
Familial partial lipodystrophy (FPL)	Cell Death-Inducing DFFA-like Effector C is a fat droplet associated protein that inhibits lipolysis.	Lack of fat in the lower extremities and metabolic disorders.	FPLD5 (#615238)	CIDEC (#612120)
	Hormone-sensitive lipase has a central role in the lipolysis of fat in adipocytes.	Lack of fat in the lower extremities and metabolic disorders.	FPLD6 (#615980)	LIPE (#151750)
Wiedemann-Rautenstrauch syndrome	RNA Polymerase III subunit C1 is the largest subunit of RNA Polymerase III	Characterized by short stature, generalized absence of fatty tissue and progeria.	WDRTS (#264090)	POLR3A (#614258)
Néstor-Guillermo progeria syndrome	Barrier-to-autointegration factor 1 dislocates lamin A from the nuclear envelope.	Generalized lipodystrophy with disorders of bone metabolism.	NGPS (#614008)	BANF1 (#603811)
Ruijs-Aalfs syndrome	SprT-like N-terminal domain protein is part of the DNA repair system.	Generalized lipodystrophy with hepatocellular carcinoma.	RJALS (#616200)	SPRTN (#616086)
Cockayne syndrome	Excision Repair Cross-Complementing Group 8 is part of the DNA repair system.	Generalized lipodystrophy with neurodegenerative disorders.	CSA (#216400)	ERCC8 (#609412)
	Excision Repair Cross-Complementing Group 6 is part of the DNA repair system.	Generalized lipodystrophy with neurodegenerative disorders.	CSB (#133540)	ERCC6 (#609413)

**Table 2:** Classification of autosomal dominant forms of lipodystrophy.

Type	Pathophysiology	Clinical Appearance	Phenotype (OMIM)	Gene (OMIM)
Familial partial lipodystrophy (FPL)		Lack of fat on the lower and upper extremities, buttocks and abdomen and metabolic disorders.	FPLD1, Kobberling (#608600)	Unknown
	Lamin A and C are nuclear lamina proteins.	Lack of fat on the lower and upper extremities, buttocks and abdomen and metabolic disorders.	FPLD2, Dunnigan (#151660)	LMNA (#150330)
	Peroxisome proliferator-activated receptor gamma is a central transcription factor in adipocyte differentiation.	Lack of fat in the lower and upper extremities and metabolic disorders.	FPLD3 (#604367)	PPARγ (#601487)
	Perilipin is a hormonally regulated phosphoprotein that is located at the fat droplets.	Lack of fat in the lower extremities and metabolic disorders.	FPLD4 (#613877)	PLIN1 (#170290)
	Caveolin is an integral part of the caveolae.	Atypical partial lipodystrophy with cataract and spasms in lower extremities.	FPLD7 (#606721)	CAV1 (#601047)
	Protein kinase B beta is a central signal protein downstream of the insulin receptor.	Lack of fat in the lower extremities and metabolic disorders.	ACT2-coupled lipodystrophy (# 240900)	ACT2 (#164731)
Hutchinson-Gilford progeria syndrome	Lamin A and C are nuclear lamina proteins.	Characterized by short stature, low body weight, generalized lack of fatty tissue and progeria.	HGPS (#176670)	LMNA (#150330)
SHORT syndrome	Phosphatidylinositol 3-kinase, Regulatory Subunit 1 (p85) is part of phosphatidylinositol 3-kinase and is a key protein in the cellular signal extension of insulin.	Dwarfism with partial absence of fatty tissue.	SHORT (#269880)	PIK3R1 (#171833)
Mandibular hypoplasia	Polymerase delta 1 encodes the catalytic subunit of DNA polymerase delta.	Absence of subcutaneous fatty tissue and metabolic abnormalities.	MDPL (#615381)	POLD1 (#174761)
Keppen-Lubinsky syndrome	Phosphatidylinositol 3-kinase, Regulatory Subunit 1 (p85) is part of phosphatidylinositol 3-kinase and is a key protein in the cellular signal extension of insulin.	Generalized lipodystrophy with disorders in psychomotor development.	KPLBS (#614098)	KCNJ6 (#600877)
Marfan Lipodystrophie syndrome	Fibrillin is a major component of the extracellular matrix.	Generalized lipodystrophy with growth abnormalities.	MFLS (#616914)	FBN1 (#134797)
